# Supplementary material for: Single Cell Transcriptomic and Chromatin Profiles Suggest Layer Vb Is the Only Layer With Shared Excitatory Cell Types in the Medial and Lateral Entorhinal Cortex
Source: Front Neural Circuits. 2022 Jan 26;15:806154. doi: 10.3389/fncir.2021.806154 (PMC8826650; doi:10.3389/fncir.2021.806154)
Supplement: Supplementary file 14 [file Data_Sheet_1.ZIP › Deep_vs_Sup_Motifs/homerResults/motif4.similar.html]

motif4

## Information for motif4

C
G
A
T
A
G
C
T
G
A
T
C
C
T
G
A
A
G
T
C
C
T
G
A
A
G
T
C
A
G
C
T
  
Reverse Opposite:  

T
C
G
A
T
C
A
G
A
G
C
T
A
C
T
G
G
A
C
T
C
T
A
G
C
T
G
A
G
C
T
A
  

|  |  |
| --- | --- |
| p-value: | 1e-73 |
| log p-value: | -1.695e+02 |
| Information Content per bp: | 1.812 |
| Number of Target Sequences with motif | 3338.0 |
| Percentage of Target Sequences with motif | 35.90% |
| Number of Background Sequences with motif | 8875.1 |
| Percentage of Background Sequences with motif | 27.25% |
| Average Position of motif in Targets | 337.2 +/- 254.4bp |
| Average Position of motif in Background | 327.1 +/- 256.2bp |
| Strand Bias (log2 ratio + to - strand density) | 0.0 |
| Multiplicity (# of sites on avg that occur together) | 1.21 |
| Motif File: | file (matrix) reverse opposite |

### Similar de novo motifs found

|  |  |  |  |  |  |  |  |
| --- | --- | --- | --- | --- | --- | --- | --- |
| Rank | Match Score | Redundant Motif | P-value | log P-value | % of Targets | % of Background | Motif file |
| 1 | 0.725 | C G A T A C G T G A T C G A C T A T G C C G T A G T A C G A T C G A C T A G C T A C G T A G C T | 1e-67 | -155.967663 | 24.87% | 17.64% | motif file (matrix) |
| 2 | 0.721 | C G T A T C A G T G C A C G A T G C T A G C A T G A T C G C T A G T A C G C T A A G T C G C A T | 1e-51 | -118.490491 | 24.50% | 18.19% | motif file (matrix) |
| 3 | 0.724 | C G T A A G C T A C T G A C G T A C T G C G A T A C T G C G T A | 1e-40 | -93.906859 | 11.85% | 7.84% | motif file (matrix) |
| 4 | 0.773 | C G T A C A T G A T C G A T C G G C A T A C T G C A G T A C T G G T C A C T G A | 1e-30 | -69.681298 | 7.37% | 4.65% | motif file (matrix) |
| 5 | 0.790 | C A T G A G C T G C A T G T A C C G T A A T G C C G T A A G T C C G A T A C G T C A T G C T A G | 1e-26 | -60.970979 | 3.39% | 1.74% | motif file (matrix) |
| 6 | 0.796 | A C T G A C G T A C T G A C G T A C T G C G T A C G T A A C T G A C G T C G T A | 1e-3 | -8.947888 | 0.29% | 0.13% | motif file (matrix) |
| 7 | 0.651 | A C G T C G T A A C G T A C G T A C G T A G T C A C G T A G T C C G T A A G T C | 1e-1 | -2.950806 | 0.35% | 0.26% | motif file (matrix) |
